# Utilizing the Intrinsic Thermal Instability of Swedenborgite Structured YBaCo_4_O_7+δ_ as an Opportunity for Material Engineering in Lithium-Ion Batteries by Er and Ga Co-Doping Processes

**DOI:** 10.3390/ma14164565

**Published:** 2021-08-14

**Authors:** Sanghyuk Park, Kwangho Park, Ji-Seop Shin, Gyeongbin Ko, Wooseok Kim, Jun-Young Park, Kyungjung Kwon

**Affiliations:** 1 Department of Energy & Mineral Resources Engineering, Sejong University, Seoul 05006, Korea; shpark@sejong.ac.kr (S.P.); radsfl@naver.com (G.K.); kevin5588@naver.com (W.K.); 2 HMC, Department of Nanotechnology and Advanced Materials Engineering, Sejong University, Seoul 05006, Korea; beongae430@naver.com (K.P.); sjs93623@nate.com (J.-S.S.)

**Keywords:** Li-ion battery, cathode material, swedenborgite, co-doping, phase stability

## Abstract

We firstly introduce Er and Ga co-doped swedenborgite-structured YBaCo_4_O_7+δ_ (YBC) as a cathode-active material in lithium-ion batteries (LIBs), aiming at converting the phase instability of YBC at high temperatures into a strategic way of enhancing the structural stability of layered cathode-active materials. Our recent publication reported that Y_0.8_Er_0.2_BaCo_3.2_Ga_0.8_O_7+δ_ (YEBCG) showed excellent phase stability compared to YBC in a fuel cell operating condition. By contrast, the feasibility of the LiCoO_2_ (LCO) phase, which is derived from swedenborgite-structured YBC-based materials, as a LIB cathode-active material is investigated and the effects of co-doping with the Er and Ga ions on the structural and electrochemical properties of Li-intercalated YBC are systemically studied. The intrinsic swedenborgite structure of YBC-based materials with tetrahedrally coordinated Co^2+^/Co^3+^ are partially transformed into octahedrally coordinated Co^3+^, resulting in the formation of an LCO layered structure with a space group of *R-3m* that can work as a Li-ion migration path. Li-intercalated YEBCG (Li[YEBCG]) shows effective suppression of structural phase transition during cycling, leading to the enhancement of LIB performance in Coulombic efficiency, capacity retention, and rate capability. The galvanostatic intermittent titration technique, cyclic voltammetry and electrochemical impedance spectroscopy are performed to elucidate the enhanced phase stability of Li[YEBCG].

## 1. Introduction

Recent research for cathode-active materials in the field of lithium-ion batteries (LIBs) pursues high energy density with a long cycle life, which has been triggered by the worldwide growth of the electric vehicle (EV) market. Ni-rich layered cathode-active materials, such as LiNi_1−x−y_Mn_x_Co_y_O_2_ (NMC) and LiNi_1−x−y_Co_x_Al_y_O_2_ (NCA), have received a great deal of attention due to their higher energy density with moderate cycling stability in the wide range of operating conditions compared to other types of cathode-active materials with olivine- (LiFePO_4_) or spinel- (LiMn_2_O_4_) structures [1]. The well-known LiCoO_2_ (LCO), which has the same layered crystal structure as NMC (space group: *R-3m*) and has been widely used mainly in portable IT devices and power tools in recent decades, became a minor option as EV battery cathode material owing to its limited practical capacity characteristics. Specifically, LCO undergoes severe structural distortion constraining the practical capacity when cycled above 4.2 V vs. Li/Li^+^ [2]. Thus, a proper cathode active material for EV requires a high Ni content in its chemical composition to satisfy high energy density despite the detrimental effect of the Ni ions on structural and thermal stability. Accordingly, many researchers have made a variety of efforts to find new dopants or surface coating compounds to suppress the severe structural degradation of Ni-rich layered cathode active materials during cycling, notwithstanding the role of Mn and Al species for structural stabilization in NMC and NCA, respectively [3,4,5,6,7,8,9,10,11,12,13,14]. This is mainly due to a specific phase transition region from H2 to H3 at around 4.2 V vs. Li/Li^+^, which leads to abrupt lattice contraction causing particle pulverization induced by microcracks [15,16].

On the other hand, Co-containing swedenborgite-structured YBaCo_4_O_7+δ_ (YBC) materials with significantly large oxygen-storage capacity are considered to be promising catalysts in solid oxide fuel cells (SOFC) working at intermediate temperatures [17,18]. It is well known that the swedenborgite structure (space group: *P6_3_mc*) of YBC with two layers of CoO_4_ tetrahedral coordinates suffers from severe phase instability by thermal decomposition at 600–800 °C due to the preference of Co ions for octahedral coordination, which makes its application in SOFC difficult [19,20]. Manthiram et al. reported that YBC doped with the optimum content of Ga, which substitutes for Co sites, can effectively overcome this phase instability at high temperatures of 600–800 °C [21]. Our recent work reported that Er and Ga co-doped YBC oxide (YEBCG) showed excellent phase stability compared to YBC, presenting long-term durability under reversible protonic ceramic cell conditions [22]. Meanwhile, there are a few reports adopting Er or Ga as dopants for cathode-active materials in LIBs. While Er-doped LiFePO_4_ and LiNi_0.5_Mn_1.5_O_4_ showed improved cycling stability [23,24], Ga-doped layered LiN_0.6_Co_0.2_Mn_0.2_O_2_ presented enhanced electrochemical performance and thermal stability, which is in line with the Manthiram group’s result based on the YBC material related to the improved phase stability at high temperatures [21,25].

Inspired by these results, we herein introduced Er and Ga co-doped swedenborgite-structured YBC as a cathode-active material in LIBs, aiming at converting the issue of phase instability of YBC at high temperatures into a challenge to explore a way towards enhancing the structural stability of layered cathode-active materials. The high temperature (800 °C) calcination process for Li-ion intercalation into the swedenborgite structure did not decompose the intrinsic swedenborgite structure of YBC and YEBCG, and forms merely a few secondary phases induced by partial thermal decomposition and a layered LCO phase, which locates Co in octahedral coordinates. The feasibility of the LCO phase, which is derived from the swedenborgite-structured YBC-based materials, as a layered cathode-active material for the LIBs was investigated, and the effects of co-doping with the Er and Ga ions on the structural and electrochemical properties of Li-intercalated YBC were studied in detail.

## 2. Experimental Section

### 2.1. Synthesis and Characterization of Materials

To prepare YBC and YEBCG powders, stoichiometric Ba(NO_3_)_2_ (99.95%, metal basis Alfa Aesar), Er(NO_3_)_3_·5H_2_O (99.9%, Alfa Aesar), Y(NO_3_)_3_·6H_2_O (99.9%, Alfa Aesar), (NH_4_)_2_Ce(NO_3_)_6_ (99.99%, Alfa Aesar), and Ga(NO_3_)_3_·xH_2_O (99.9%, metal basis, Alfa Aesar) were dissolved in distilled water with glycine as a combusting fuel and then heated on a heating plate (MSH-20D, DAIHAN Scientific Co., Ltd., Wonju, Korea) at 350 °C until the metal nitrates converted into black ashes. After the combustion, the ashes were ground with a mortar and then calcined at 1000 °C for 12 h under an air atmosphere to obtain a single-phased crystalline [22]. In order to identify the crystal structure of the YBC-based materials, an X-ray diffraction (XRD) technique (X’Pert, PANalytical, Cu Kα radiation, Almelo, The Netherlands) was carried out with a step size of 0.026° in a 2θ range from 10 to 80°. Utilizing the XRD data, the FullProf software was used for Rietveld refinement to yield lattice parameters. The morphological characterization of the YBC-based materials was performed using a field emission scanning electron microscope (FE-SEM, SU-8010, Hitachi Ltd., Tokyo, Japan) with energy-dispersive X-ray spectroscopy (EDS). To measure the Brunauer-Emmett-Teller (BET) surface area, nitrogen gas was used for an adsorption/desorption method (BELSORP-max, BEL Inc., Leitchfield, KY, USA) at 77 K to remove residual impurities and moisture with an average mass of 0.54 g for each specimen. For the sake of Li-intercalation, the synthesized YBC and YEBCG were mixed with LiOH‧H_2_O in a molar ratio of 1:1.1, followed by calcination under air atmosphere for 10 h at 800 °C. The Li-intercalated YBC and YEBCG oxides are referred to as Li[YBC] and Li[YEBCG], respectively.

### 2.2. Electrochemical Analysis

For a cathode slurry, the Li[YBC] and Li[YEBCG] cathode active materials, carbon black (Super-P) as a conducting material, and polyvinylidene fluoride (KF 1100) as a binder in a weight ratio of 80:10:10 were thoroughly mixed in a N-methylpyrrolidinone (NMP) solution with 30% solid content and casted on Al foil as a current collector. Electrochemical properties were investigated using CR2032-type coin cells, which were fabricated in a moisture-controlled glove box under an argon atmosphere. While 1 M LiPF_6_ dissolved in a mixture of ethyl methyl carbonate and ethylene carbonate (2:1, *v*/*v*) was used as an electrolyte, Li metal foil and polyethylene film were used as anode and separator, respectively. Cycling stability was assessed by cycling the cells at 0.1 C (46 mA g^−1^ as 1 C) in different potential ranges of 2.0 to 4.5 V for 50 cycles and 2.5 to 4.3 V for 100 cycles, while a rate capability test was conducted in the potential range from 2.0 to 4.5 V in various C-rate conditions using a battery cycler (WBCS3000L, WonAtech Ltd., Seoul, Korea) at 25 °C. For activating the cells, cycling at 0.1 C during initial two cycles was conducted prior to the electrochemical tests as a formation step. Electrochemical impedance spectroscopy (EIS) and cyclic voltammetry (CV) were performed using a potentiostat system (versaSTAT 3, AMETEK Inc., Berwyn, PA, USA) to compare the change in internal cell resistance and the degree of overpotentials during cycling, respectively. A galvanostatic intermittent titration technique (GITT) was conducted to calculate Li-ion diffusion coefficients. All the values of potentials were based on Li/Li^+^ in this study unless otherwise mentioned.

## 3. Results and Discussion

**Figure 1:**
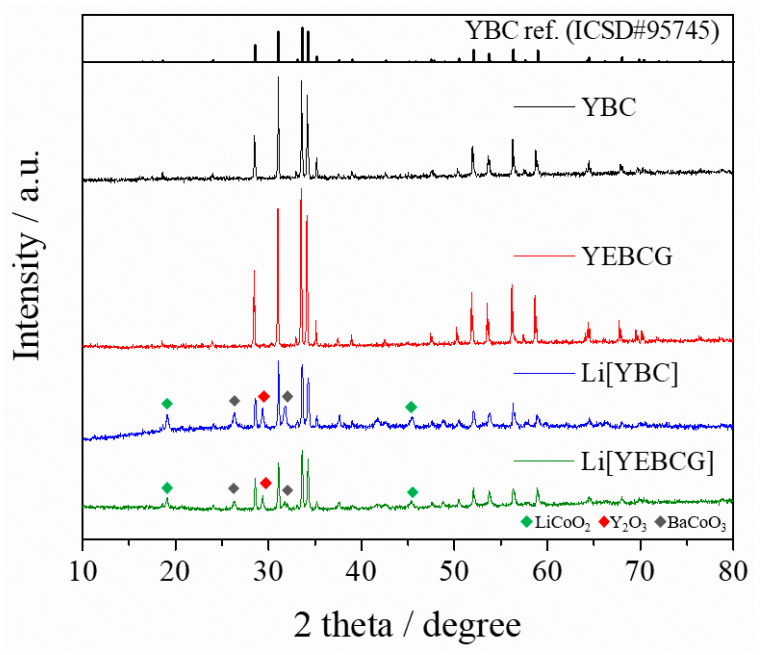
XRD patterns of pristine YBC and YEBCG, and their Li-intercalated forms of Li[YBC] and Li[YEBCG].

The XRD patterns of swedenborgite-structured YBC and YEBCG before and after the Li-ion intercalating calcination process at 800 °C are presented in Figure 1. It is known that BaCoO_3−__δ_ and Y_2_O_3_ secondary phases can form readily due to oxidative thermal decomposition from the intrinsic YBC structure above 600 °C [26]. This structural degradation of Li[YEBCG] induced by the thermal decomposition was mitigated when Er and Ga were co-doped in the YBC structure. As seen in Figure 1, the peak intensity of secondary phases was reduced for Li[YEBCG], indicating the better structural stability at the oxidative high temperature than Li[YBC]. A LiCoO_2_ phase allowing the (de)intercalation of Li ions was also newly formed (marked as green-colored symbols in the figure), which might be ascribed to the evolution of Co ions located at octahedral coordinates during the calcination process. Likewise, the Co ions in BaCoO_3_ (marked as gray-colored symbols in the figure), which is a main decomposition product of YBC, have octahedral coordination [27], whereas the Co ions in YBC have a tetrahedral coordination. Meanwhile, Table 1 lists the crystal lattice parameters of pristine YBC and YEBCG, showing the increased lattice parameters of a- and c-axes with larger lattice volume for YEBCG compared to YBC, which is consistent with the previous literature [22].

**Figure 2:**
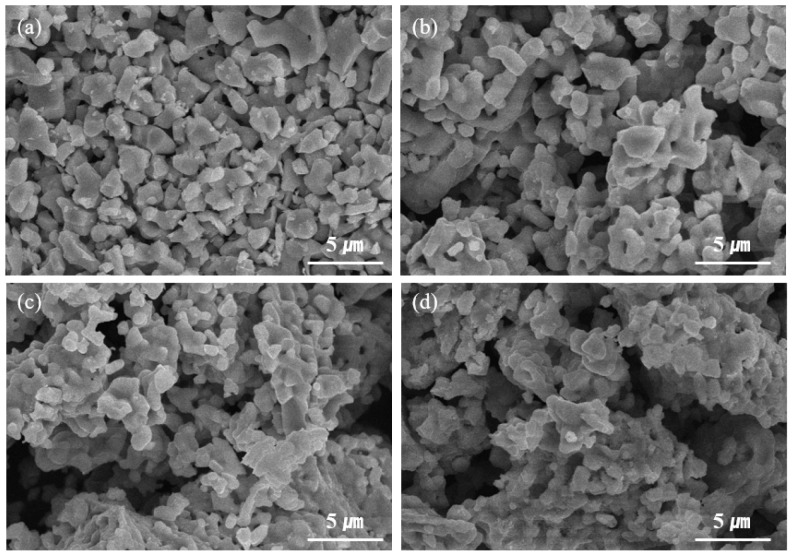
FE-SEM images of (**a**) YBC, (**b**) Li[YBC], (**c**) YEBCG, and (**d**) Li[YEBCG].

**Figure 3:**
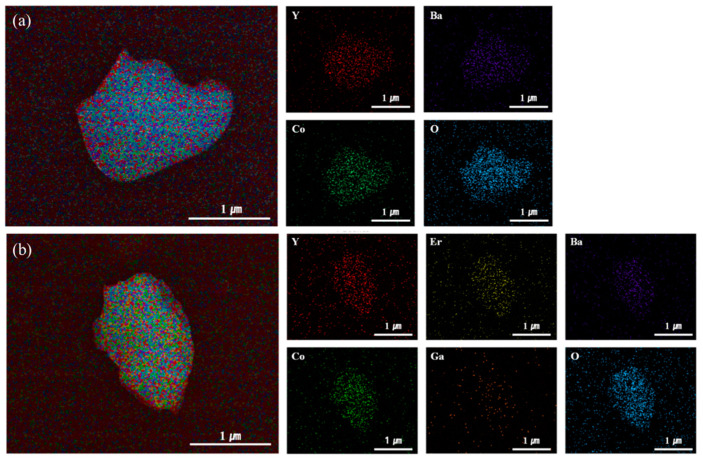
Elemental mapping images of (**a**) Li[YBC] and (**b**) Li[YEBCG] analyzed by EDS.

To elucidate the relationship between changes in structural phase and morphology before and after the Li-intercalation process, a FE-SEM analysis was performed. Figure 2 shows the surface morphology of YBC, Li[YBC], YEBCG, and Li[YEBCG]. Compared to the pristine particles, the Li-intercalated Li[YBC] and Li[YEBCG] samples exhibited a negligible change in morphology. It can be concluded that the aforementioned phase deformation induced by the oxidative thermal decomposition does not affect the morphological change from their original shapes. Meanwhile, the elemental mapping data of Li[YBC] and Li[YEBCG] are presented, as shown in Figure 3a for Li[YBC] and Figure 3b for Li[YEBCG], respectively, indicating that all of constituent elements were evenly distributed.

**Figure 4:**
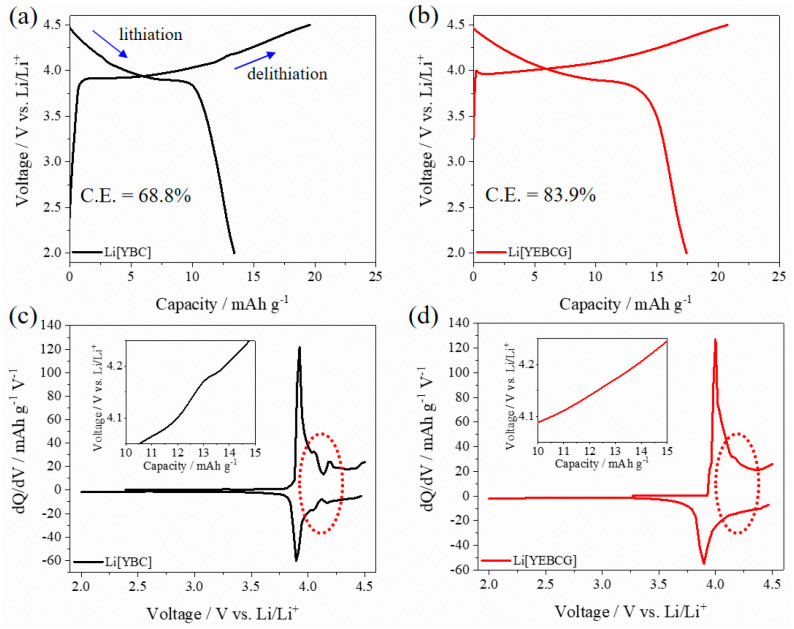
(**a**,**b**) Initial charge/discharge and (**c**,**d**) differential capacity curves of Li[YBC] and Li[YEBCG], respectively. A distinct phase transition region (red-dotted circles in (**c**,**d**)) in the potential around 4.2 V was displayed with insets of the corresponding voltage profile. (C.E.: Coulombic efficiency).

Figure 4a,b display the initial charge (delithiation) and discharge (lithiation) curves of Li[YBC] and Li[YEBCG] cathode active materials. Li[YEBCG] showed increased charge/discharge capacities with enhanced Coulombic efficiency compared to Li[YBC]. A differential capacity analysis was performed in order to look into the phase transition behavior during the initial cycle in a potential range of 2.0–4.5 V, as shown in Figure 4c,d, which was derived from the initial charge/discharge curves. Two minor peaks between 4.05 and 4.25 V were observed in Li[YBC] besides the major peaks at around 3.9 V, whereas the phase transition was effectively suppressed in the case of Li[YEBCG], indicating the enhanced structural stability. These electrochemical behaviors of Li[YBC] and Li[YEBCG] are consistent with the typical phase transition behavior of the LCO cathode active materials in LIBs [2]. Because these phase transitions during a repetitive cycling, specifically above 4.2 V, can bring about the deterioration of LIB performance, the mitigation of the phase transition is a crucial issue for layered cathode active materials [15]. Consequently, LIB performance including the cycle life and the rate capability of Li[YEBCG] is expected to outperform Li[YBC] considering the structural and electrochemical features of enlarged lattice volume and suppressed phase stability.

**Figure 5:**
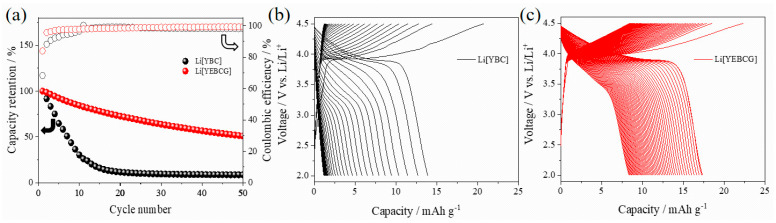
(**a**) Capacity retention at 0.1 C with Coulombic efficiency, and corresponding voltage profiles of (**b**) YBC and (**c**) YEBCG during 50 cycles.

Figure 5 presents the cycle performance of the cathode active materials for 50 cycles at 0.1 C in a potential range of 2.0–4.5 V. The capacity retention of Li[YBC] was drastically aggravated right after the initial cycle and reached less than 10% after about 20 cycles. By contrast, Li[YEBCG] showed superior capacity retention with a relatively stable feature of Coulombic efficiency over the entire cycling process. After 50 cycles, Li[YEBCG] had a capacity retention of 50%, which was about ten times higher than Li[YBC]. The corresponding voltage profiles of Li[YBC] (see Figure 5b) clearly showed rapid capacity fading with increasing overpotentials during cycling, while Li[YEBCG] presented a feature of mitigated capacity fading (see Figure 5c), indicating the positive effects of co-doping with Er and Ga on the suppression of the undesired phase transition as aforementioned in Figure 4. Long-term cycle performance was additionally conducted in the potential range of 2.5–4.3 V in Figure S1, which is closer to the practical operating condition of LIBs including a constant voltage charging step with a moderate charging cut-off potential. The long-term cycle performance showed relatively enhanced cycling stability compared to the 4.5 V cut-off condition resulting from a limited (de)lithiation range, which led to decreased discharge capacities for both samples. In general, a higher upper cut-off potential shows higher capacities at the expense of cycling stability [28].

**Figure 6:**
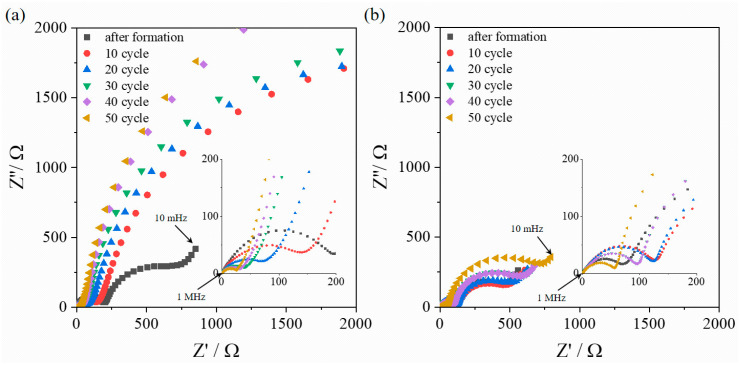
Nyquist plots of (**a**) Li[YBC] and (**b**) Li[YEBCG] with increasing cycle numbers.

In order to electrochemically elucidate a specific reason for the improved capacity retention of Li[YEBCG], the EIS was conducted in a frequency range from 0.01 Hz to 1 MHz with an alternating voltage amplitude of 15 mV. The EIS technique in LIBs is considered as one of the most attractive analytical tools that can separate and quantify cell resistance in-situ, avoiding any impact of moisture or oxygen on sensitive samples [29]. Figure 6 shows the resulting Nyquist plots of Li[YBC] and Li[YEBCG] with an increasing cycle number under a pseudo-equilibrium state for charged cells. Li[YBC] has shown extremely larger impedance growth than Li[YEBCG] after the formation step. Noticeably, the first semicircles in a high frequency region that are related to the film resistance induced by the formation of solid electrolyte interphase (SEI) between electrodes and electrolytes gradually shrank with an increased cycle number from 10 to 50 in both samples (see the insets in the figures). It is hard to intuitively interpret the abnormal phenomenon about the gradual decrease of film resistance, which is contrary to other relevant research [30,31]. In the meantime, the second semicircles in a low frequency region regarding charge transfer resistance (R_ct_) indicated incremental trends in both samples during cycling due to the structural degradation of the cathode active materials. In addition, this might correlate with the intrinsically weak adhesion property of YBC-based materials to current collectors (see Figure S2). Therefore, it can be concluded that the poor capacity retention of Li[YBC] was caused by severe impedance growth in the cell, while the doping with Er and Ga is deemed to be a crucial factor to reinforce the host structure and facilitate the charge transfer process in the Li[YEBCG] structure.

**Figure 7:**
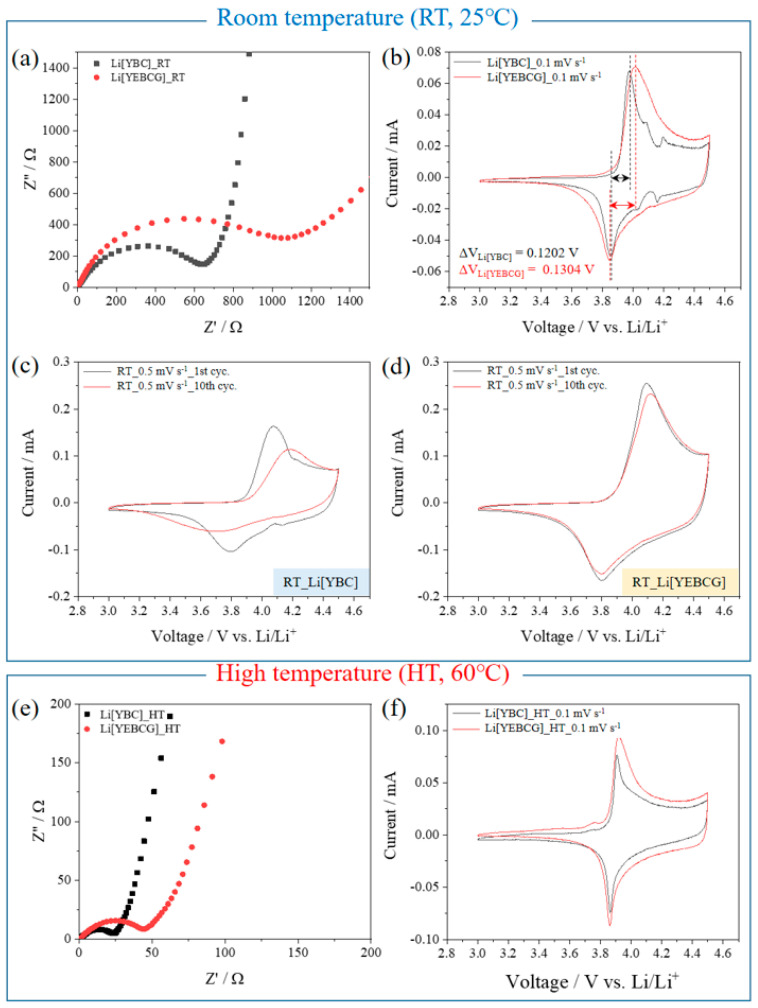
Nyquist plots and CV curves of Li[YBC] and Li[YEBCG] under various experimental conditions. (**a**,**e**) EIS data before the CV tests at equilibrium states, (**b**,**f**) initial CV curves at 0.1 mV s^−1^, and (**c**,**d**) CV curves during the 1st and 10th cycles at 0.5 mV s^−1^, where (**a**–**d**) were tested at 25 °C and (**e**,**f**) at 60 °C.

Through a series of EIS and CV measurements under various electrochemical conditions, the superior electrochemical performance of Li[YEBCG] compared to that of Li[YBC] was clearly confirmed again. As seen in Figure 7a, the EIS results obtained at equilibrium states before the following CV tests showed that Li[YBC] has a relatively lowered R_ct_ value than that of Li[YEBCG], which leads to a slightly reduced potential intervals (Δ*V*) between main anodic and cathodic peaks compared with Li[YEBCG] as shown in Figure 7b. Although the Δ*V* generally indicates the reversibility of Li-ion (de)intercalation, the obvious feature of the suppressed phase transition of Li[YEBCG] during the cycling at 0.1 mV s^−1^ positively affected the repetitive CV measurements at 0.5 mV s^−1^ as shown in Figure 7c,d. Li[YBC] presented a different electrode polarization induced by severe overpotentials after 10 cycles leading to the increase in Δ*V*, whereas the degree of overpotentials for Li[YEBCG] was slightly changed after 10 cycles representing the enhancement of structural stability of Li[YEBCG] by the Er and Ga co-doping process. It is worth noting that the R_ct_ value of Li[YEBCG] measured after the formation step became smaller than Li[YBC] (see Figure 6).

The EIS and CV measurements were additionally conducted at a different temperature of 60 °C, as displayed in Figure 7e,f, respectively. Compared with the results at 25 °C, the R_ct_ values have drastically decreased in both samples, which might be attributed to the effect of elevated temperature facilitating the migration of Li^+^ ions in the bulk structure. In the CV data, the overpotentials of both samples were also reduced. Interestingly, the feature of severe phase transition of Li[YBC] during the CV measurement at 0.1 mV s^−1^ completely disappeared, which could lead to the improvement of structural stability during the cycling. Although the capacity retention of Li[YEBCG] at 60 °C was indeed improved in comparison with that at 25 °C, the drastic aggravation of capacity retention after the initial cycle was still a problematic issue (see Figure S3).

**Figure 8:**
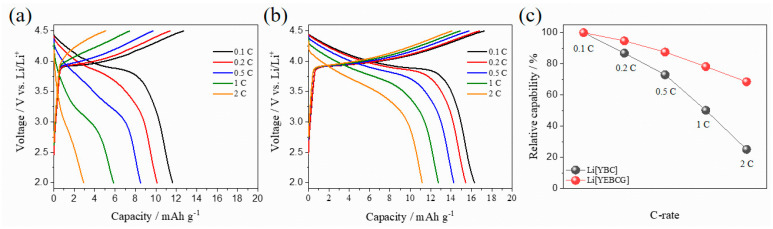
Rate capability of (**a**) Li[YBC] and (**b**) Li[YEBCG], and (**c**) their relative capability at various discharging C-rates from 0.1 to 2 C with a fixed charging C-rate of 0.1 C.

The rate capability tests of Li[YBC] and Li[YEBCG] were performed to look into the kinetic behavior during reversible (de)lithiation reactions at different discharging C-rates with a fixed charging C-rate of 0.1 C, as shown in Figure 8a,b. Whereas the overpotentials of Li[YBC] rapidly increased along with the decrease in operating voltage as the C-rate increases, Li[YEBCG] presented a much better rate performance than Li[YBC]. Moreover, given that the decay tendency in the charge capacity of Li[YEBCG] was apparently mitigated compared with Li[YBC], it can be understood that the intrinsic swedenborgite structure of Li[YEBCG] is effectively stabilized by the co-doping with Er and Ga. Figure 8c shows the relative capability of the samples with respect to discharge capacities at various C-rates compared to the discharge capacity at 0.1 C. Li[YEBCG] had superior relative capability to that of Li[YBC] as the C-rate increases, also suggesting that the Er and Ga co-doped host structure of YEBCG is favorable for Li ions to migrate into the bulk structure.

**Figure 9:**
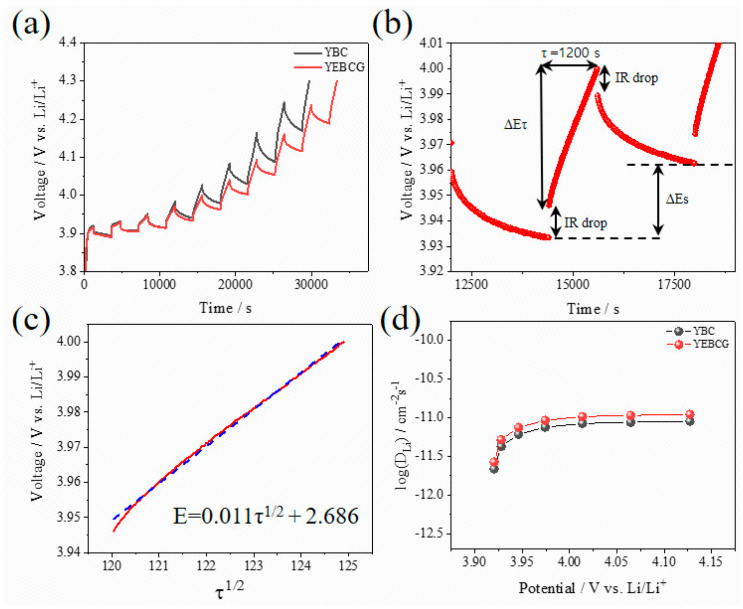
(**a**) GITT curves of Li[YBC] and Li[YEBCG] during the second charge process at a current density of 4.6 mA g^−1^. (**b**) GITT curve at a single titration step of Li[YEBCG] with (**c**) corresponding linear behavior in the relationship of *E* vs. *τ*^1/2^. (**d**) Li-ion diffusion coefficients of Li[YBC] and Li[YEBCG] calculated from the GITT curves as a function of the cell potential.

Lastly, to compare the Li-ion diffusion coefficients (*D*_Li_^+^) between the samples, a GITT analysis was carried out as shown in Figure 9. GITT curves shown in Figure 9a were obtained during the second charging process, and a single titration step was described in Figure 9b, where ∆*E_s_* (V) is the voltage change between steady states and ∆*E_τ_* (V) is the total change of cell voltage in a single titration step [32]. Fick’s second law is utilized to calculate the *D*_Li_^+^ as below [33]:

(1) $$D_{{\mathrm{Li}}^{+}}=\frac{4}{\pi }{\left(\frac{mV}{MS}\right)}^{2}{\left(\frac{\Delta E_{S}}{\tau \left(\frac{dE_{\tau }}{d\sqrt{\tau }}\right)}\right)}^{2}\;\left(\tau \ll \frac{L^{2}}{D_{{\mathrm{Li}}^{+}}}\right)$$

As seen in Figure 9c, under the assumption that *E* vs. $\sqrt{\tau }$ shows a straight-line behavior during the titration step, Equation (1) can be simplified into Equation (2) [34,35],

(2) $$D_{{\mathrm{Li}}^{+}}=\frac{4}{\pi \tau }{\left(\frac{mV}{MS}\right)}^{2}{\left(\frac{\Delta E_{S}}{\Delta E_{\tau }}\right)}^{2}\left(\tau \ll \frac{L^{2}}{D_{{\mathrm{Li}}^{+}}}\right)$$

where *m* is the mass (g), *V* is the molar volume (cm^3^ mol^−1^), and *M* is the molecular weight (g mol^−1^) of cathode active materials. *S* is the contact area (cm^2^) between electrodes and electrolytes, and *L* is the Li-ion diffusion length (cm).

The *D*_Li_^+^ values of samples calculated using Equation (2) were plotted as a function of cell potential, showing relatively higher *D*_Li_^+^ values of Li[YEBCG] compared to Li[YBC] over the entire potential range (Figure 9d). It can be explained that Li[YEBCG] has better kinetic behavior for the (de)intercalation of Li ions compared to Li[YBC] regardless of C-rate conditions due to the higher *D*_Li_^+^. Therefore, the comprehensively improved electrochemical properties of Li[YEBCG] are clearly attributed to the effect of cationic substitution of Er and Ga that stabilize the host structure from undesired phase transition.

## 4. Conclusions

We synthesized Er and Ga co-doped swedenborgite-structured YBC, which was utilized as a cathode-active material to investigate the potential enhancement of phase stability. The peak intensity of secondary phases in the XRD patterns was mitigated in the case of Li[YEBCG], indicating the better structural stability at the oxidative high temperature compared with Li[YBC]. A LiCoO_2_ phase allowing the (de)intercalation of Li ions was also newly formed, which might result from the evolution of Co ions located at octahedral coordinates during the calcination process. The partial thermal deformation resulting from oxidative calcination barely affected the morphologies and their elemental distribution of YBC and YEBCG. Regarding the LIB performance, Li[YEBCG] exhibited superior capacity retention with enhanced charge/discharge capacities compared to Li[YBC], showing the mitigated feature of phase transition at around 4.2 V. The poor capacity retention of Li[YBC] was ascribed to severe impedance growth during cycling, while the doping with Er and Ga was considered to be a crucial factor to reinforce the host structure and facilitate the charge transfer process in the Li[YEBCG] structure, leading to the effective suppression of impedance growth. Whereas the overpotentials of Li[YBC] rapidly increased along with the decrease in operating voltage as the C-rate increased, Li[YEBCG] showed much better rate performance than Li[YBC]. The calculated *D*_Li_^+^ of samples showed the relatively higher *D*_Li_^+^ values of Li[YEBCG] compared to Li[YEBCG] over the entire potential range, explaining that Li[YEBCG] has better kinetic behavior for the (de)intercalation of Li ions than Li[YBC] regardless of C-rate conditions. Consequently, the comprehensively improved electrochemical properties of Li[YEBCG] are clearly attributed to the enhanced phase stability induced by the stabilizing effect of Er and Ga co-doping on the host structure from the undesired phase transition. Future work will include the influence of Er and Ga co-doping in more conventional LIB cathode active materials on the mitigation of structural degradation during cycling.

## Figures and Tables

**Table 1:** Crystallographic parameters of YBC and YEBCG calculated by Rietveld refinement.

	YBC	YEBCG
a-axis (Å)	6.2993	6.3097
c-axis (Å)	10.2534	10.2674
Volume (Å^3^)	352.3540	354.0059

## Data Availability

The data presented in this study are available on request from the corresponding author.

## Supplementary Materials

The following are available online at https://www.mdpi.com/article/10.3390/ma14164565/s1, Figure S1: Initial charge/discharge curves and long-term cycle performance of Li[YBC] and Li[YEBCG] in a potential range of 2.5–4.3 V, Figure S2: Comparative pictures of (a) Li[YBC] and (b) Li[YEBCG] after the cycling test, Figure S3: Cycle performance of Li[YBC] at different temperatures of 25 and 60 °C.
