# Supplementary material for: Utilizing the Intrinsic Thermal Instability of Swedenborgite Structured YBaCo4O7+δ as an Opportunity for Material Engineering in Lithium-Ion Batteries by Er and Ga Co-Doping Processes
Source: Materials (Basel). 2021 Aug 14;14(16):4565. doi: 10.3390/ma14164565 (PMC8400947; doi:10.3390/ma14164565)
Supplement: Supplementary file 1 [file materials-14-04565-s001.zip › materials-1316402-supplementary.pdf]

Supplementary Information

Utilizing the Intrinsic Thermal Instability of Swedenborgite Structured $YBaCo_4O_{7+\delta}$ as an Opportunity for Material Engineering in Lithium-Ion Batteries by Er and Ga Co-Doping Processes

Sanghyuk Park [1,†], Kwangho Park [2,†], Ji-Seop Shin [2], Gyeongbin Ko [1], Wooseok Kim [1], Jun-Young Park [2,*] and Kyungjung Kwon [1,*]

[1] Department of Energy & Mineral Resources Engineering, Sejong University, Seoul 05006, Korea; shpark@sejong.ac.kr (S.P.); radsfl@naver.com (G.K.); kevin5588@naver.com (W.K.)

[2] HMC, Department of Nanotechnology and Advanced Materials Engineering, Sejong University, Seoul 05006, Korea; beongae430@naver.com (K.P.); sjs93623@nate.com (J.-S.S.)

* Correspondence: jyoung@sejong.ac.kr (J.-Y.P.); kfromberk@gmail.com (K.K.); Tel.: +82-2-3408-3947 (K.K.)

† These authors contributed equally to this work.

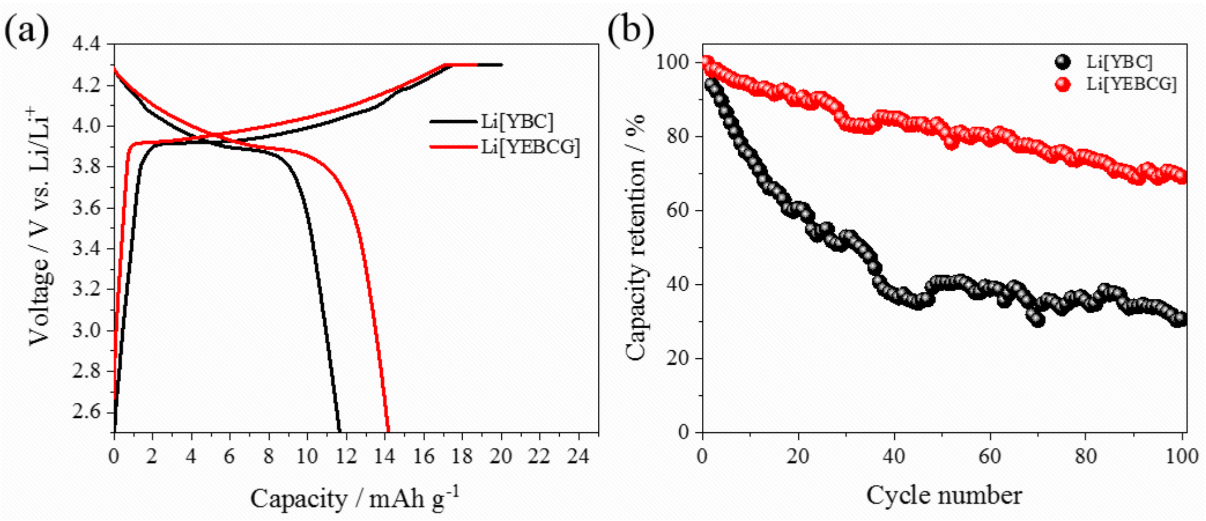

Figure S1. (**a**) Initial charge/discharge curves and (**b**) long-term cycle performance of Li[YBC] and Li[YEBCG] in a potential range of 2.5–4.3 V.

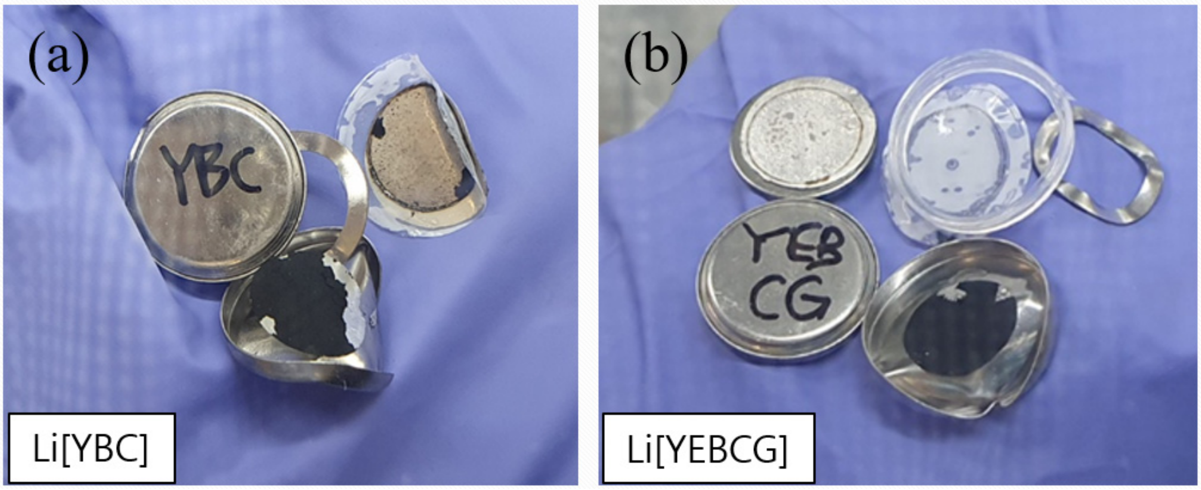

Figure S2. Comparative pictures of (**a**) Li[YBC] and (**b**) Li[YEBCG] after the cycling test, representing intrinsically weak adhesion property of Li[YBC] with current collectors.

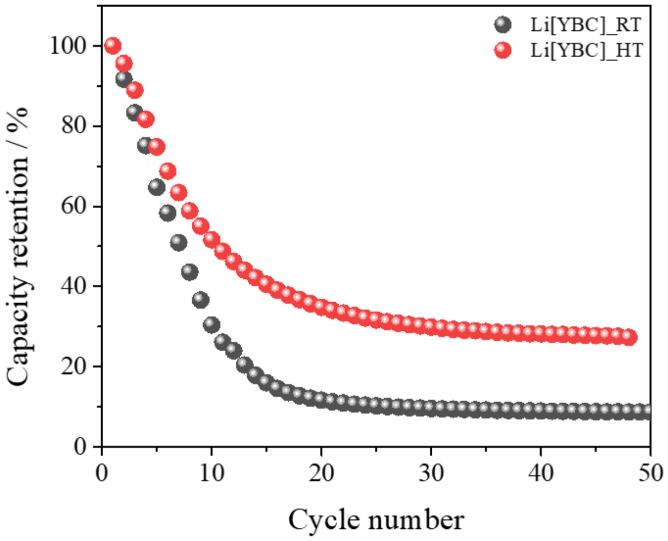

Figure S3. Cycle performance of Li[YBC] at different temperatures of 25 and 60℃.
